# Prehistoric Trepanning

**Published:** 1893-02-25

**Authors:** 


### PREHISTORIC TREPANNING.

We furnish a brief résumé of the able article by Dr. Robert Munro in the *Fortnightly Review* on this interesting subject. The first person to call attention to the fact that trepanning had been practised in prehistoric ages was Dr. Prunières, of the town of Marvejol (Lozère), who found a perforated skull in his researches among the dolmens in his neighbourhood some twenty years ago. Dr. Paul Broca subsequently devoted much time and study to the subject, and added greatly to the facts already known by his researches in the artificial caves newly brought to light in the valley of Petit Morin. The people who lived and died in these caves belonged to the neolithic period. He discovered that the polished portions round the margin of some of the perforations were due to cicatricial deposits, the result of pathological action in living bodies, and formed the following conclusions: 1. During the neolithic period a surgical operation was practised which consisted in making an opening through the skull for the treatment of certain internal maladies. 2. The skulls of those individuals who survived this trepanning were considered to be possessed of particular properties of a mystic order, and when such individuals died rondelles or fragments were often cut from their skulls, which were used as amulets. It was found that these perforations were performed chiefly, though not exclusively, on children. Skulls operated upon in this manner have been found in such widely distant localities as the Island of Bute and the Yuca Cemetery in the Valley of Ynca, Peru, showing the existence of a very general belief in the virtue of trepanning. It appears to be in use at this day among the South Sea Islanders and natives of Montenegro for even trifling ailments. Dr. Broca considered it could have been performed only by scraping the bone with a sharp piece of flint till a perforation was effected. The exact site of the operation was undetermined, any part of the head seems to have been selected on the body of a bone, or across the suture. The object of trepanning was probably to cure epilepsy, convulsions, lunacy, or diseases ascribed to the agency of an evil spirit. The following are examples from the neolithic stations of the Marne districts: 1. The skull of a young person (brachycephalic) shows an oval perforation over the right lambdoidal suture measuring 1⅝ by 1⅜ of an inch. There is no trace of cicatrisation, therefore the patient must have died under the operation, or it was a post-mortem one. 2. The skull of an aged person had a perforation in the left parietal bone about an inch behind the coronal suture. The lesion had completely cicatrised. 3. The skull of an aged person had been twice trepanned. The patient had survived both operations. The two perforations were close together on the left parietal bone, one was very large, measuring 2⅛ by 1⅞ inches.

